# First Detection of *Rocahepevirus* in Urban Wastewater from Guinea: A One Health Alert

**DOI:** 10.3390/pathogens15040385

**Published:** 2026-04-03

**Authors:** Bakary Doukouré, Yann Le Pennec, Cissé Fatoumata, Ramatoulaye Diallo, Issiaga Touré, Noël Tordo, Pierre Roques

**Affiliations:** 1 Institut Pasteur de Guinée, Conakry BP 4416, Guinea; lpnc.yann@gmail.com (Y.L.P.); fatoumata.cisse@pasteur-guinee.org (C.F.); ramadalla90@gmail.com (R.D.); issiaga.toure@pasteur-guinee.org (I.T.); ntordo@pasteur.fr (N.T.); pierre.roques@pasteur.fr (P.R.); 2 Institute for Tropical Medicine, University of Tübingen, 72074 Tübingen, Germany; 3 Immune Diseases, Microbiology and Innovative Therapies (IDMIT/UMRS 1184), Université Paris-Saclay, Inserm, CEA, 92265 Fontenay-aux-Roses, France; 4 Virology Department, Institut Pasteur, 75015 Paris, France

**Keywords:** *Rocahepevirus ratti*, RHEV, urban wastewater, phylogenetic analysis, zoonotic risk

## Abstract

Hepatitis E virus (HEV) is a major cause of acute viral hepatitis worldwide, with zoonotic genotypes detected in humans and animals. In Africa, limited data exist on environmental HEV circulation. Here, we report the first detection of *Rocahepevirus ratti* (RHEV) in urban wastewater from Conakry, Guinea. From December 2024 to April 2025, *Rocahepevirus ratti* (RHEV) has been detected in 35 out of 180 urban untreated wastewater samples in Conakry, Guinea. The phylogenetic analysis of partial HEV ORF1 genome segments reveals clustering with African rodent RHEV strains, highlighting environmental contamination and potential zoonotic risk for human populations in proximity. This finding underscores the need for integrated One Health surveillance to monitor HEV transmission at the human–animal–environment interface in West Africa, particularly in Guinea.

## 1. Introduction

Hepatitis E virus (HEV), or *Paslahepevirus balayani*, is a major cause of acute viral hepatitis worldwide and is transmitted through fecal–oral routes. While specific human genotypes exist, zoonotic genotypes are also emerging from animal reservoirs with potential risks for human health. Zoonotic HEV genotype 3, mainly transmitted by pigs, has led to outbreaks in humans in Africa. HEV-3 circulation has been confirmed in Guinean pigs both by seroprevalence studies and the molecular characterization of HEV 3c (OR283252.1) in feces as well as in water effluent from pigs’ enclosures (PX441305) [1]. Beyond *Paslahepevirus balayani,* other hepeviruses belonging to distinct taxonomic groups circulate in animal populations. Although data on the environmental circulation of hepeviruses in Africa remain limited [2], *Rocahepevirus ratti* (RHEV), primarily circulating in rodents, has recently been detected [3]. It is occasionally associated with human infections [4,5]. There is currently no centralized wastewater treatment plant or structured sewage management system, and beyond humans, numerous rats and the free circulation of pigs between open enclosures can be seen. Thus, the aim of this study was to investigate the presence of HEV in the urban wastewater of the Conakry area, as part of a One Health surveillance program from the Institut Pasteur de Guinée for the ANSS (Agence National de Sécurité Sanitaire).

## 2. Materials and Methods

Between December and April 2025, grab raw wastewater samples (RWWSs) of 200 mL were collected manually from 10 quarters in Conakry using sterile polypropylene bottles once a week. Immediately after collection, the samples were transported to the laboratory at 4 °C to preserve their integrity. Sample processing was carried out within a maximum of three days post-collection. RNA that could not be assessed using RT-PCR or RT-qPCR within this timeframe was stored at −80 °C to preserve viral RNA integrity prior to analysis. Freeze–thaw cycles were minimized to reduce potential effects on RNA detection.

For viral detection, RWWSs were first clarified by centrifugation (3000 rpm/30 min) and then were concentrated by polyethylene glycol (PEG 8000) precipitation. Briefly, 4 g of PEG 8000 (10%) and 0.9 g of NaCl (0.4 M) were added to 40 mL of clarified wastewater. The mixtures were stirred until fully dissolved and then incubated overnight at 4 °C. After incubation, the samples were centrifuged for 2 h at 12,000× *g*; the supernatant was discarded, and the pellet was resuspended in 500 µL of PBS. The obtained concentrates were aliquoted and stored at −80 °C until RNA extraction. Viral RNA was extracted from 140 µL of concentrated wastewater samples (CWWSs) using the QIAamp Viral RNA Mini Kit (Qiagen, Hilden, Germany), following the manufacturer’s instructions. To account for the possible co-circulation of genetically distinct hepeviruses in environmental samples, the same RNA extracts were systematically screened using two genus-specific molecular assays.

First, *hepevirus*-specific HEV RNA detection was performed using an RT-qPCR targeting ORF2/3 [6], and positive samples were further confirmed by nested RT-PCR assays targeting ORF1 and ORF2 [7]. Then, the RNA extracts were screened using a *Rocahepevirus*-specific nested RT-PCR targeting ORF1 [8]. The primers used for RT-qPCR and nested RT-PCR assays are summarized in Supplementary Table S1, including sequences and target genomic regions. To definitively confirm viral identity and exclude cross-reactivity between assays, ORF1 amplicons from positive samples were subjected to sequencing using primers specific for either Rocahepevirus or Paslahepevirus. PCR products were purified and quantified using the Qubit system (Thermo Fisher Scientific, Illkirch, France). Libraries were then prepared according to the manufacturer’s instructions and loaded onto a flow cell for sequencing using the Oxford Nanopore Technologies MinION platform (Oxford, UK). Raw signal data were demultiplexed through barcode assignment and subjected to basecalling. FASTA-format sequences were generated using the ARTIC nCoV bioinformatics pipeline. The resulting FASTA files were processed with a workflow adapted from the ARTIC protocol to generate consensus sequences. These consensus sequences were deposited in GenBank AC# PX408741-PX408746.

Finally, Hepatitis E virus Genotyping Tools (software version 2.22.2, tool version 1.16) were used to identify mutations and assign each sequence to the corresponding HEV variant.

## 3. Results

**Figure 1:**
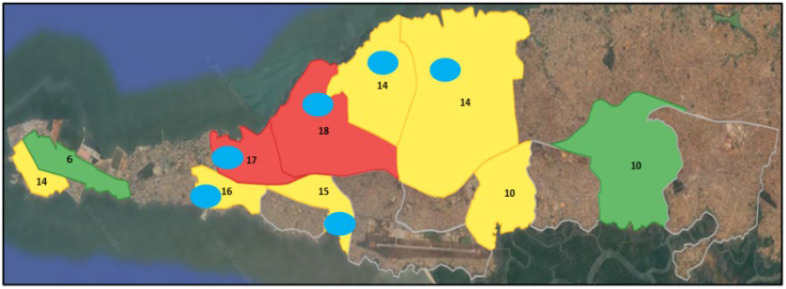
Spatial distribution of HEV detection sites in Conakry wastewater area. For each site, the number of weeks (out of 18) during which HEV RNA was detected is indicated. The color coding highlights the sites where high viral load was detected frequently (about 11 weeks) in **red**, moderately (1–4 weeks) in **yellow**, or at lower viral load in **green**. The **blue** circles indicate the origin of the sequenced samples. Base map © 2024 Google Earth.

Of 180 water samples, 135 (75%) were positive using ORF3 Paslahepevirus-specific RT-qPCR. Positive RT-qPCR signals were detected at all 10 sampling sites over the study period. Ct values varied between sites, with lower Ct values observed at some locations (Ct >32), whereas other sites showed higher Ct values (Ct < 32). Figure 1 illustrates the spatial distribution of these sampling sites and the frequency of HEV detection across the 18-week monitoring period.

Thirty-five of the 135 positive samples were confirmed using ORF1-nested RT-PCR [7] and none using ORF2-nested RT-PCR. These 35 samples were also positive using ORF1-nested RT-PCR specific for *Rocahepevirus ratti* [8].

Following ORF1-nested RT-PCR amplification, 12 positive samples were subjected to sequencing. Within the pipeline, either HEV-3h strain or RHEV strain V-105 genome was used as reference (JQ013794.1 and JX120573, respectively). From this read processing, only the *Rocahepevirus* reference sequence provided consensus sequences of ±900 pb. From the 12 samples sequenced on the 10 sites, we retained six representative samples of the different areas at different time points from December to April.

The sequencing performance of all samples aligned to HEV reference JX120573.1 is summarized in Table 1, showing read numbers, coverage, and average base and mapping qualities.

Reference sequence**:** HEV, GenBank accession JX120573.1. Num reads: Number of sequencing reads aligned to this region. Coverage bases: Number of bases covered by at least one read. Coverage: Average number of reads covering each base (or proportion of covered bases). Mean depth: Average sequencing depth across the region. Mean baseq: Mean base quality score (Phred scale), indicating confidence in base calls. Mean mapq: Mean mapping quality score, indicating confidence in read alignment to the reference.

**Figure 2:**
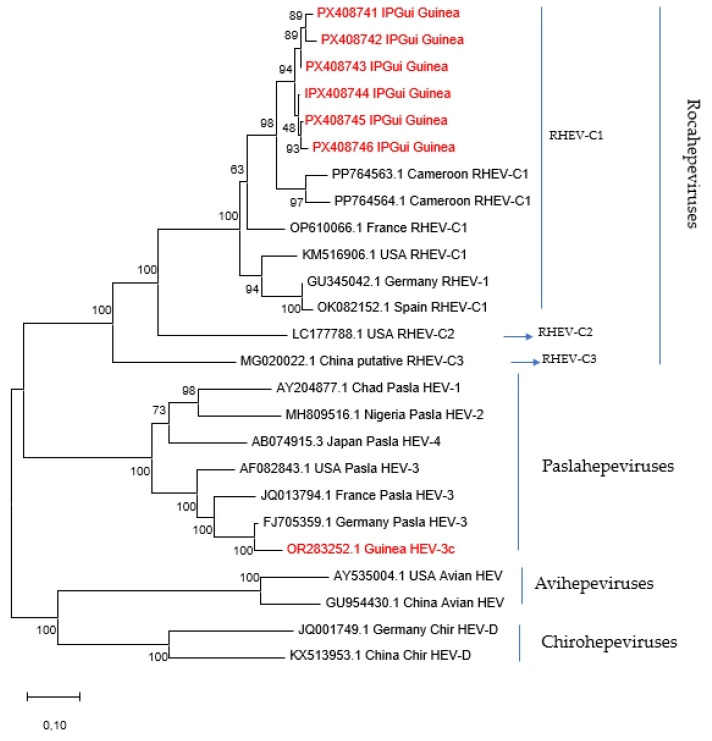
Phylogenetic tree of the Hepeviridae family based on 900 nucleotides of the ORF1 gene, constructed using the Neighbor-Joining method with 500 bootstrap replicates in MEGA version 12 (https://www.megasoftware.net accessed on 27 June 2025). Six HEV sequences in Guinean wastewater and the HEV found in a Conakry pig are in red. Numbers correspond to the bootstrap percentage supporting each node.

A phylogenetic analysis based on these 900 nucleotide fragments of the ORF1 region showed that the Conakry wastewater sequences clustered within the sub-genotype RHEV-C1 of *Rocahepevirus ratti* (Figure 2). The Conakry’s sequences (accession numbers: PX408741-PX408746) clustered with HEV rodent sequences from Cameroon (GenBank PP764563.1-PP764564.1) [3] with a high bootstrap value (98%).

## 4. Discussion/Conclusions

This is the first report of *Rocahepevirus ratti* RNA (RHEV-C1) circulation in Conakry wastewater. As *Paslahepevirus* of genotype 3c (OR283252.1) was detected in pig feces and several effluent streams in 2023 in Conakry (PX441305) [1], we first tested wastewater samples with *Paslahepevirus*-specific RT-PCR and confirmed 75% (135/180) of positive samples during the whole study period (4 months). However, we did not find any HEV-3 *Paslahepevirus*-positive samples. Interestingly, 35 samples were also positive with *Rocahepevirus*-specific RT-PCR and were further identified by NGS as the RHEV-C1 strain. These results suggest that *Rocahepevirus ratti* circulated in a diffuse and prolonged way during our 4 months of screening, with hot spots suggesting the local concentration of rat populations; long-term circulation dynamics require further longitudinal and quantitative studies.

Partial ORF1 sequencing was selected because this genomic region is commonly used for HEV classification and phylogenetic analysis in environmental surveillance studies [3]. Similar approaches have been used in recent wastewater-based HEV monitoring studies in Africa [3]. The clustering of Guinean sequences with rodent-associated RHEV strains from Cameroon supports the circulation of this virus lineage in Africa.

The sequences obtained from wastewater samples that were positive with *Rocahepevirus ratti*-specific RT-PCR were included in a phylogenetic analysis alongside reference sequences from the Hepeviridae family. Care was taken to include representative sequences from *Rocahepevirus* and *Paslahepevirus* lineages to illustrate the relationship between the detected strains and previously described hepeviruses. As expected, sequences from HEV *Paslahepevirus* found in Guinean pigs were not included in the same clade as *Rocahepevirus ratti* (Figure 2).

The detection of *Rocahepevirus ratti* in wastewater from Conakry reflects a heterogeneous spatial distribution. Areas where the virus was detected more frequently correspond to sectors that have less favorable sanitary conditions, potentially promoting the presence of rodents, which are *Rocahepevirus* reservoirs. These rodents often inhabit peri-domestic areas, facilitating frequent contact between humans, animals, and the environment. This pattern illustrates the One Health concept, emphasizing the interconnectedness of human, animal, and environmental health. This environmental contamination generates a potential risk of cross-species transmission to humans and/or pigs [9]. Despite the scarce information available, this risk must be assessed in Africa like it is in Asia (8) and Europe [10]. An integrated surveillance gathering regular information on rodent and swine reservoirs as well as related human exposure seems mandatory in West Africa, particularly in Guinea.

It is important to note that, while the data provide insights into the spatial distribution of *Rocahepevirus ratti* in Conakry wastewater, any interpretation regarding continuous circulation or transmission should be made with caution. Further genomic characterization would be required to fully resolve the taxonomic diversity of hepeviruses circulating in wastewater samples, and additional surveys in humans or the trapping of rats are required to evaluate the true risk associated with this virus circulation. From a public health perspective, the detection of *Rocahepevirus ratti* in wastewater highlights the importance of environmental surveillance as an early warning system. These findings support the implementation of integrated One Health monitoring strategies combining environmental sampling, rodent surveillance, and the investigation of potential human exposure. Strengthening sanitation infrastructure and urban rodent control programs may also help reduce potential zoonotic transmission risks in rapidly growing cities such as Conakry.

## Figures and Tables

**Table 1:** Sequencing coverage and quality metrics of the six sequenced samples aligned to HEV reference JX120573.

Sequ ID	Numreads	Coverage Bases	Coverage	Mean Depth	Mean Baseq	Mean Mapq
PX408741	1697	777	77.08	1259.63	29.5	34.4
PX408742	1158	794	78.76	861.16	29.7	24.7
PX408743	528	775	76.88	391.72	29.4	25.7
PX408744	265	775	76.88	198.41	29.2	25.8
PX408745	510	780	77.38	379.33	29.3	28
PX408746	738	776	76.98	547.92	29.4	28.6

## Data Availability

Data supporting the findings of this study are available from the corresponding author, B.D., on request.

## Supplementary Materials

The following supporting information can be downloaded at https://www.mdpi.com/article/10.3390/pathogens15040385/s1. Supplementary Table S1. Primer sequences (5′–3′), target regions, and references for RT-qPCR and nested RT-PCR assays used for HEV detection.

## Abbreviations

The following abbreviations are used in this manuscript:

ARTIC	Arctic Network protocol for pathogen sequencing
Ct	Cycle threshold
CWWS	Concentrated wastewater sample
FASTA	Format for nucleotide/amino-acid sequence files
HEV	Hepatitis E virus
HEV-3	Hepatitis E virus genotype 3
RHEV-C1	*Rocahepevirus ratti* (rat HEV) genotype C1
NGS	Next-generation sequencing
ORF	Open reading frame
ORF1/ORF2/ORF3	Open reading frame regions 1, 2, and 3
PEG	Polyethylene glycol
qPCR/RT-qPCR	Quantitative reverse-transcription polymerase chain reaction
PCR/RT-PCR/nested RT-PCR	(Nested) reverse-transcription polymerase chain reaction
RNA	Ribonucleic acid
RWWS	Raw wastewater sample
