# Supplementary material for: First Detection of Rocahepevirus in Urban Wastewater from Guinea: A One Health Alert
Source: Pathogens. 2026 Apr 3;15(4):385. doi: 10.3390/pathogens15040385 (PMC13119015; doi:10.3390/pathogens15040385)
Supplement: Supplementary file 1 [file pathogens-15-00385-s001.zip › pathogens-4225743-supplementary.pdf]

Type of RT-PCR	Target	Forward sequences	Reverse sequences	Probe sequences	Reference
RT-qPCR	ORF2/3	5'-GGTGGTTTCTGGGGTGAC-3'	5'-AGGGGTTGGTTGGATGAA-3'	5'-TGATTCTCAGCCCTTCGC-3'	(6)
Nested RT PCR	ORF1 (Outer)	5'-GAGGCYATGGTSGAGAARG-3'	5'-GCCATGTTCCAGACRGTRTTCC-3'		(7)
	ORF1 (Inner)	5'-GGTTCCGYGCTATTGARAARG-3'	5'-TCRCCAGAGTGYTTCTTCC-3'		(7)
	ORF2 (Outer)	5'-CCGACGTCYGTYGAYATGAA-3'	5'-TTRTCCTGCTGAGCRTTCTC-3´		(7)
	ORF2 (Inner)	5'-AAGTGAGCGCCTACAYTAYCG-3'	5'-CTCGCCATTGGCTGAGAC-3'		(7)
Nested RT PCR *Rocahepevirus*	ORF1 (Outer)	5'-CTTGGTTYAGGGCCATAGAG-3'	5'-CAGCAGCGGCACGAACAGCA-3'		(8)
	ORF1 (Inner)	5'-TTYAGGGCCATAGAGAAGGC-3'	5'-ACAGCAAAAGCACGAGCACG-3'		(8)

Supplementary Table S1. Primer sequences (5'–3'), target regions, and references for RT-qPCR and nested RT-PCR assays used for HEV detection.
